# *Brassica* vegetables—an undervalued nutritional goldmine

**DOI:** 10.1093/hr/uhae302

**Published:** 2024-10-30

**Authors:** Xiaomeng Zhang, Qiong Jia, Xin Jia, Jie Li, Xiaoxue Sun, Leiguo Min, Zhaokun Liu, Wei Ma, Jianjun Zhao

**Affiliations:** State Key Laboratory of North China Crop Improvement and Regulation, Key Laboratory of Vegetable Germplasm Innovation and Utilization of Hebei, Collaborative Innovation Center of Vegetable Industry in Hebei, College of Horticulture, Hebei Agricultural University, No. 2596 Lekai South Street, Lianchi District, Baoding, Hebei 071000, China; State Key Laboratory of North China Crop Improvement and Regulation, Key Laboratory of Vegetable Germplasm Innovation and Utilization of Hebei, Collaborative Innovation Center of Vegetable Industry in Hebei, College of Horticulture, Hebei Agricultural University, No. 2596 Lekai South Street, Lianchi District, Baoding, Hebei 071000, China; State Key Laboratory of North China Crop Improvement and Regulation, Key Laboratory of Vegetable Germplasm Innovation and Utilization of Hebei, Collaborative Innovation Center of Vegetable Industry in Hebei, College of Horticulture, Hebei Agricultural University, No. 2596 Lekai South Street, Lianchi District, Baoding, Hebei 071000, China; Department of Biochemistry and Metabolism, John Innes Centre, Norwich Research Park, Norwich NR4 7UH, United Kingdom; State Key Laboratory of North China Crop Improvement and Regulation, Key Laboratory of Vegetable Germplasm Innovation and Utilization of Hebei, Collaborative Innovation Center of Vegetable Industry in Hebei, College of Horticulture, Hebei Agricultural University, No. 2596 Lekai South Street, Lianchi District, Baoding, Hebei 071000, China; State Key Laboratory of North China Crop Improvement and Regulation, Key Laboratory of Vegetable Germplasm Innovation and Utilization of Hebei, Collaborative Innovation Center of Vegetable Industry in Hebei, College of Horticulture, Hebei Agricultural University, No. 2596 Lekai South Street, Lianchi District, Baoding, Hebei 071000, China; Vegetable Research Institute, Suzhou Academy of Agricultural Sciences, No. 2351 Dongshan Avenue, Linhu Town, Wuzhong District, Suzhou, Jiangsu 215155, China; State Key Laboratory of North China Crop Improvement and Regulation, Key Laboratory of Vegetable Germplasm Innovation and Utilization of Hebei, Collaborative Innovation Center of Vegetable Industry in Hebei, College of Horticulture, Hebei Agricultural University, No. 2596 Lekai South Street, Lianchi District, Baoding, Hebei 071000, China; State Key Laboratory of North China Crop Improvement and Regulation, Key Laboratory of Vegetable Germplasm Innovation and Utilization of Hebei, Collaborative Innovation Center of Vegetable Industry in Hebei, College of Horticulture, Hebei Agricultural University, No. 2596 Lekai South Street, Lianchi District, Baoding, Hebei 071000, China

## Abstract

The genus *Brassica* includes six species and over 15 types of vegetables that are widely cultivated and consumed globally. This group of vegetables is rich in bioactive compounds, including glucosinolates, vitamins (such as vitamin C, folate, tocopherol, and phylloquinone), carotenoids, phenols, and minerals, which are crucial for enriching diets and maintaining human health. However, the full extent of these phytonutrients and their significant health benefits remain to be fully elucidated. This review highlights the nutrient compositions and health advantages of *Brassica* vegetables and discusses the impacts of various processing methods on their nutritional value. Additionally, we discuss potential strategies for enhancing the nutrition of *Brassica* crops through agronomic biofortification, conventional breeding, and biotechnological or metabolic engineering approaches. This review lays the foundation for the nutritional improvement of *Brassica* crops.

## Introduction

**Figure 1:**
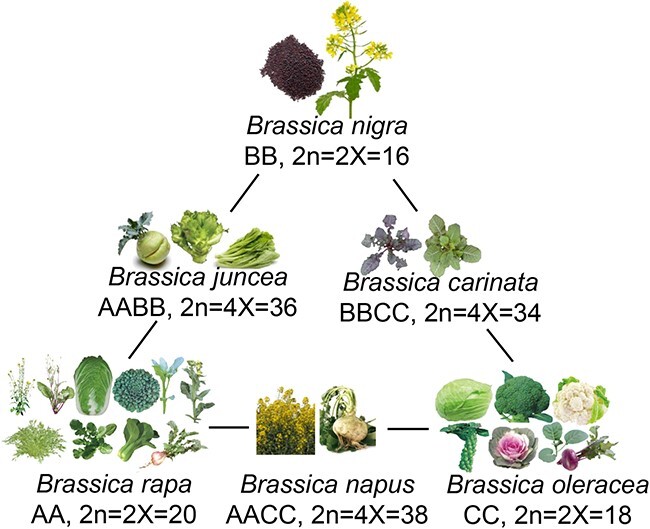
U’s triangle composed of *Brassica* crops. The images of *Brassica rapa* and *Brassica oleracea* were taken from [1]; *Brassica carinata* from [3]; baby mustard (*Brassica juncea* var. gemmifera) from [4]; and rutabaga (*Brassica napus*) from Britannica, 2023 (https://www.britannica.com/plant/rutabaga); Other images were modified according to those in the VEER website (https://www.veer.com).

The *Brassica* genus, belonging to the *Brassicaceae* family, comprises six economically important crop species: three diploids—*Brassica nigra* (BB genome), *Brassica rapa* (AA genome), and *Brassica oleracea* (CC genome)—and three allotetraploids—*B. juncea* (AABB genome), *Brassica carinata* (BBCC genome), and *Brassica napus* (AACC genome) [1, 2] (Fig. 1). These species, collectively referred to as the “U’s triangle” [5], originated from common ancestors ~24 million years ago and were among the first plants domesticated by humans [6]. *Brassicas* are primarily classified into three categories based on the parts consumed: oilseeds, vegetables, and condiments (Table 1). The vegetable category primarily includes *B. juncea* (mustard greens), *B. napus* (rutabaga), and *B. rapa* (Chinese cabbage, turnip, mizuna, tatsoi, winter rape, and pak choi), as well as *B. oleracea* (broccoli, kohlrabi, kale, cauliflower, Brussels sprouts, cabbage, and cabbage mustard). Oilseeds predominantly comprise *B. napus* (rapeseed), and condiments typically utilize seeds from *B. juncea* and *B. nigra*. Given its agricultural and economic significance, *Brassica* is among the most extensively cultivated and consumed crop groups worldwide. Its production has been expanding over the past decades. In 2022, the production of major *Brassica* crops—including cabbages, cauliflower, broccoli, mustard seeds, and rape or colza seeds—totaled over 186 million tons, covering ~44 million hectares, with a combined value exceeding 92 billion US dollars (FAOSTAT, 2022) (www.fao.org/faostat/zh). The genus’s significant horticultural relevance has fueled extensive molecular research, uncovering that the genomes of cultivated *Brassica* species have undergone considerable triplications and chromosomal rearrangements, thus driving their evolution and development [8–11].

*Brassica* crops contribute significantly to human health through dietary intake. As plant-based foods, *Brassica* vegetables are excellent sources of macronutrients essential for daily nutritional needs (Supplementary Table 1). Some of their metabolites are also closely linked to sensory attributes, such as taste, aroma, and pungency [12–14]. Although most *Brassica* crops, apart from those used for oil production, are not primary sources of calories due to their low carbohydrate and lipid content, they provide a diverse range of macronutrients, including amino acids, organic acids [13, 15], proteins, soluble sugars, and dietary fibers [16–18] (Supplementary Table 1). Increasing evidence suggests that consuming *Brassica* vegetables may reduce the risk of chronic diseases, especially various types of cancer, owing to their unique health-promoting phytonutrients [19–22]. Thus, increasing the intake of *Brassica* vegetables offers a practical and natural method for consumers to improve their health through functional foods, rather than relying on supplements or extracts.

**Table 1:** Six *Brassica* species and their respective vegetables^a)^

*Brassica* species	Genome	Latin name	Common name	Other edible way
*Brassica rapa*	A	ssp. *pekinensis* ssp. *chinensis* ssp. *rapa* ssp. *nipposinica* ssp. *narinosa* ssp. *oleifera*	Chinese cabbage pak choi turnip mizuna tatsoi winter rape	oilseed
*Brassica nigra*	B	—	—	condiment
*Brassica oleracea*	C	var. *italica* var. *botrytis* var. *gongylodes* var. *acephala* var. *capitata* var. *gemmifera* var. *albiflora*	broccoli cauliflower kohlrabi kale cabbage brussels sprouts cabbage mustard	condiment
*Brassica napus*	AC	var. *oleifera* var. *napobrassica*	rapeseed/canola rutabaga	oilseed
*Brassica juncea*	AB	var. *multiceps* var. *gemmifera* var. *megarrhiza* var. *tumida*	leaf mustard baby mustard root mustard stem mustard	condiment
*Brassica carinata*	BC	—	—	oilseed, condiment

a) Modified from ref. [[1]; [7]]

—, Unknown vegetable or varieties

**Figure 2:**
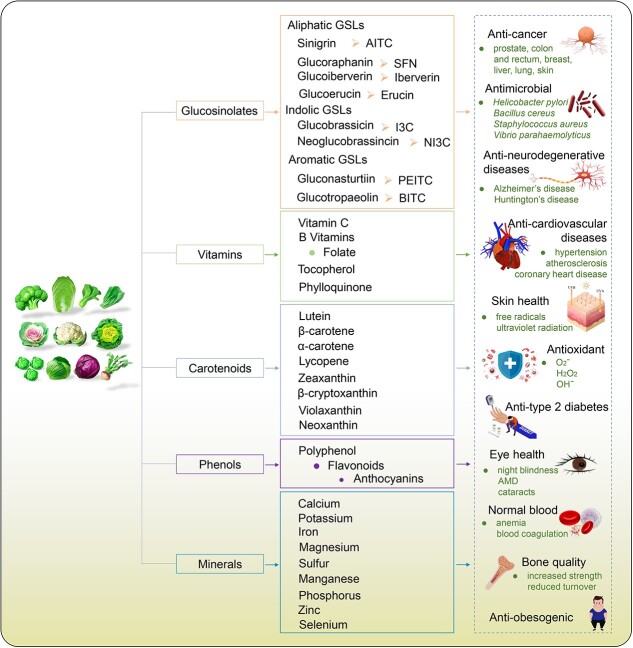
The main phytonutrients in *Brassica* vegetables and their associated health benefits. AMD, age-related macular degeneration. The images of *Brassica rapa* and *Brassica oleracea* were modified based on those of [1]. Additional images were adapted from those available on the VEER website (https://www.veer.com).

Inadequate intake of essential nutrients, particularly micronutrients, is often a consequence of imbalanced diets with low consumption of vegetables and fruits. This deficiency elevates the risk of chronic diseases such as cardiovascular diseases, type 2 diabetes, obesity, and various cancers [23–26]. Biofortification, which enhances the nutritional profile of the edible parts of crops, emerges as a notably cost-effective strategy among various methods to increase the nutritional value of staple foods. This approach has been successful in boosting the nutritional value of staple crops [27]. *Brassica* crops are naturally rich in various bioactive compounds with established health benefits, and are gaining popularity and hold significant potential for nutritional enhancement. This review delves into the bioactive nutrients (phytonutrients) of major *Brassica* crops and their roles in promoting a healthy diet (Fig. 2). Additionally, we explore biofortification and nutritional enhancement strategies through agronomic practices, conventional cross-breeding, and metabolic engineering of key micronutrients. Ultimately, this review aims to lay the groundwork for the nutritional improvement of *Brassica* crops, an underestimated nutritional goldmine.

### Phytonutrients and their health benefits

1)
**Glucosinolates and their hydrolysis products**

Glucosinolates (GSLs) are sulfur- and nitrogen-containing secondary metabolites derived from amino acids. *Brassica* vegetables such as cauliflower, Brussels sprouts, broccoli, and cabbage, are primary dietary sources of GSLs [17, 28]. These compounds exhibit considerable structural diversity, which is classified based on the amino acid precursors (Table 2). GSLs are biologically inactive until they are hydrolyzed by specific enzymes called myrosinases, which release various bioactive derivatives including isothiocyanates (ITCs), indoles, and thiocyanates [51]. Among the most noteworthy health benefits of GSLs are their anticarcinogenic properties. Research has consistently shown that consumption of *Brassica* vegetables is negatively associated with the risk of several cancers, including colorectal, prostate, gastric, and breast cancers [52–54]. The cancer-protective effects of these vegetables are primarily attributed to compounds such as sulforaphane (SFN) [55, 56], erucin [43], benzyl ITC, phenethyl ITC (PEITC) [35], indole-3-carbinol (I3C), and its biologically active dimer, 3,3′-diindolylmethane [29, 57–59], and N-methoxyindole-3-carbinol (NI3C) [46]. These bioactives are derived from the breakdown of aliphatic (glucoraphanin [GRA], glucoerucin [GER]), aromatic (glucotropaeolin [GTP], gluconasturtiin [GNS]), and indolic (glucobrassicin [GBS], neoglucobrassicin [NGBS]) GSLs [7, 46]. Additionally, allyl isothiocyanate, a product of sinigrin (SIN) hydrolysis, induces cell cycle arrest and apoptosis in drug-resistant cancer cells [32]. Iberverin, a degradation product of glucoiberverin (GIV), shows antineoplastic activities against human hepatocellular carcinoma [41]. Moreover, NI3C acts as a more effective inhibitor of human colon cancer cell proliferation compared to its precursor I3C [46], and SFN has demonstrated antibacterial activity against *Helicobacter pylori*, a major causative agent of gastric ulcers and cancer [60, 61]. Furthermore, PEITC, which is derived from turnips, exhibits antimicrobial properties against foodborne pathogens such as *Bacillus cereus*, *Staphylococcus aureus*, and *Vibrio parahaemolyticus* [48]. However, not all glucosinolate breakdown products from *Brassica* vegetables are beneficial; some can have anti-nutritional effects. For example, PRO has been reported to cause goiters and other adverse effects on animal nutrition, suggesting that individuals with thyroid disorders should limit their intake of PRO-rich vegetables [62]. Despite these concerns, there is no definitive epidemiological evidence that goitrogenic effects are a major cause of human diseases.

**Table 2:** Common and chemical names, hydrolysis products of GSLs commonly found in the family *Brassica*^b)^

Common names	Abbr.	Chemical names of R-groups	Hydrolysis products	Mainly found in	Bioactivity
*Aliphatic GSLs*					
Progoitrin	PRO	2(R)-2-Hydroxy-3-butenyl	Goitrin	Brussels sprouts, turnip	Decrease thyroid hormone production^c)^
Sinigrin	SIN	2-Propenyl	Allyl ITC	Brown mustard, cabbage	Anticancer activity; prevents obesity and insulin resistance^d)^
Glucoiberin	GIB	3-Methylsulphinylpropyl	3-Methylsulphinylpropyl ITC	Cabbage	Anticarcinogenic activity^e)^
Gluconapin	GNP	3-Buteny	3-butenyl ITC	Brussels sprouts, brown mustard, broccoli, kale	Anticarcinogenic activity^f)^
Glucoraphanin	GRA	4-Methylsulphinylbutyl	Sulforaphane	Broccoli	Anticarcinogenic activity; decrease risk of various cardiovascular diseases^g)^
Glucoraphenin	GRE	4-Methylsulphinyl-3-buteny	?	—	
Glucoiberverin	GIV	3-Methylthiopropyl	Iberverin	Cabbage, broccoli	Antineoplastic activities^h)^
Glucobrassicanapin	GBN	4-Pentenyl	4-pentenyl ITC	Broccoli, rapeseed	Antibacterial Activity^i)^
Glucoerucin	GER	4-Methylthiobutyl	Erucin	Chinese cabbage	Anticancer activity; anti-inflammatory activity^j)^
Glucoalyssin	GAL	5-Methylsulphinylpentenyl	?	—	
Gluconapoleiferin	GNL	2-Hydroxy-4-pentenyl	?	—	
Glucocheirolin	GCR	3-Methylsulfonylpropyl	?	—	
Glucoberteroin	GBT	5-Methylthiopentyl	?	Kohlrabi	
Epiprogoitrin	EPI	2(S)-2-Hydroxy-3-butenyl	?	—	
					
*Indolic GSLs*					
Glucobrassicin	GBS	3-Indolylmethyl	Indole-3-carbinol	Almost all *Brassica*s	Anticarcinogenic and antitumorigenic properties; antibacterial activity^k)^
Neoglucobrassincin	NGBS	1-Methoxy-3-indolylmethyl	N-methoxyindole-3-carbinol	Chinese cabbage, pak choi, broccoli, cauliflower	Anticancer activity^l)^
4-Methoxyglucobrassicin	4-MGBS	4-Methoxy-3-indolylmethyl	?	Chinese cabbage, cabbage	
4-Hydroxyglucobrassicin	4-OHGBS	4-Hydroxy-3-indolylmethyl	?	Broccoli	
					
*Aromatic GSLs*					
Gluconasturtiin	GNS	2-Phenylethy	Phenethyl ITC	Turnip	Anticancer activity; anti-inflammatory activity; cardio protective activity; antimicrobial activity^m)^
Glucotropaeolin	GTP	Benzyl	Benzyl ITC	—	Anticancer activity; antibacterial activity^n)^
Glucobarbarin	GBB	(S)-2-Hydroxy-2-phenylethyl	?	—	

b) Modified from ref. [[29]; [28]; [30]; [7]]

c) Ref. [31]

d) Ref. [[32]; [33]]

e) Ref. [34]

f) Ref. [[35]; [36]]

g) Ref. [[37]; [38]; [39]; [40]]

h) Ref. [41]

i) Ref. [42]

j) Ref. [[43]; [44]]

k) Ref. [45]

l) Ref. [46]

m) Ref. [[47]; [48]; [49]; [50]]

n) Ref. [[42]; [35]]

?, Unknown specific products

—, Low concentrations in *Brassica* crops

Different *Brassica* species exhibit variations in the hydrolysis products of GSLs with anti-cancer activity. For example, I3C is present in nearly all *Brassica* crops, including pak choi, Chinese cabbage, turnip, broccoli, cauliflower, cabbage, kohlrabi, kale, Brussels sprouts, and mustard green [29], whereas SFN, PEITC and erucin are predominantly found in broccoli, turnip, and Chinese cabbage, respectively [30]. Compared to Chinese cabbage “Chiifu”, pak choi “Bras”, yellow sarson Z1 (AA genome), and cabbage (CC genome), the turnip (AA genome) taproots exhibited higher levels of two health-promoting aliphatic GSLs, GRA, and GER [14]. However, the GSL content in 45 turnip varieties varies with plant earliness and habit. Specifically, one extra-late group and one late group turnip variety display the highest total GSL content, indicating their potential as valuable sources of bioactive vegetables [63]. In pak choi, GSL levels are closely related to the growth status of leaves. Compared to sprouts, total indole GSLs, GBS, and NGBS, are significantly higher in mature leaves [64]. In broccoli, the concentration of SFN, a product derived from GRA, peaks at the stage of commercial maturity [65]. The highest levels of GRA are observed at the mature head stage, with a subsequent decrease as flowering begins [66]. A study on the flowering stalk tissues of 107 global rapeseed accessions (comprising 25 spring, 34 winter, and 48 semi-winter accessions) detected seven GSL compounds, indicating continuous variation [67]. In kohlrabi, total GSL content is higher in purple varieties compared to pale green ones. Specifically, GER is found exclusively in purple kohlrabi, with concentrations four times higher in the flesh than in the skin [68]. Additionally, the oval Chinese cabbage cultivar exhibits higher levels of total GSLs, whereas the rectangular cultivar contains higher levels of GBS [69]. In conclusion, the consumption of *Brassica* vegetables provides greater cancer protection than the general consumption of fruits and vegetables due to their diverse and potent GSL profiles [70].

2)
**Vitamins**

*Brassica* vegetables are an excellent dietary source of antioxidants due to their rich composition of health-enhancing phytochemicals such as vitamin C, various B vitamins, tocopherol, and phylloquinone. Traditionally, citrus fruits have been regarded as the primary source of vitamin C. However, *Brassica* vegetables can provide up to 50% of the daily recommended intake of vitamin C, depending on dietary preferences across different nations, making them a valuable natural source of this vitamin in human diets [71]. A study examining 27 *Brassica* varieties found that the concentration of vitamin C varied from 12.9 to 176 mg per 100 g FW [72]. Consuming 100 grams of *Brassica* vegetables, such as pak choi, broccoli, cabbage, and cauliflower, can meet an adult’s daily recommended intake of 40 mg of vitamin C (Supplementary Table 1). Lower serum levels of ascorbic acid can have severe health implications, such as an increased production of reactive oxygen species, which can elevate the risk of chronic diseases and accelerate aging [73, 74]. Ascorbate, or vitamin C, serves a dual role, functioning not only as an antioxidant that protects cells from oxidative stress but also enhancing iron bioavailability. It achieves this by chelating with ferric iron in the acidic environment of the stomach [75]. Therefore, the substantial vitamin C content in *Brassica* crops significantly boosts their nutritional value, especially in terms of iron absorption. Epidemiological studies consistently show that dietary intake of vitamin C is linked to a reduced risk of cancer. Importantly, the connection between vitamin C consumption and cancer prevention is stronger when the vitamin is sourced from fruits and vegetables rather than from synthetic supplements like pills or extracts [76–80].

*Brassica* vegetables are rich in a broad spectrum of B vitamins, with the exception of vitamin B12, which cannot be synthesized by plants (Supplementary Table 1). Folate, also known as vitamin B_9_, is crucial for human nutrition, and its deficiency can lead to slow growth rates in children and anemia. High concentrations of folate are found in key leafy vegetables of this genus, including cabbage, broccoli, Chinese cabbage, pak choi, and cauliflower [81–83]. Notably, folate levels vary among different cultivars, accessions, and plant parts [83, 84].

Significant concentrations of phylloquinone, a fat-soluble vitamin also known as vitamin K1, are present in various green *Brassica* vegetables, such as broccoli, kale, green cabbage, and rapeseed [85, 86]. To meet the recommended daily intake of vitamin K, men would need to consume either 158 grams of cabbage or 183 milliliters of canola oil (from *B. napus* or *B. rapa*), whereas women would require 118 grams of cabbage or 138 milliliters of canola oil [87]. Research across all genotypes of mustard (*Brassica juncea* L.), collard (*B. oleracea* L.), and turnip (*B. rapa* L.) greens has shown that phylloquinone concentrations are consistent between younger and older leaves, with averages of 435 μg and 459 μg per 100 g FW, respectively [84]. Within these genotypes, however, notable variations exist. For instance, in collard greens, phylloquinone content ranges from 308.7 to 576.9 μg per 100 g FW in younger leaves and from 369.2 to 616.5 μg per 100 g FW in older leaves. Furthermore, turnip greens typically exhibit higher phylloquinone concentrations than most collard genotypes [84]. In choy sum (*B. rapa* ssp. *chinensis var*. *parachinensis*), phylloquinone levels increase during early growth stages—from 377 μg per 100 g FW in microgreens to 433 μg per 100 g FW in seedlings—but decrease to 363 μg per 100 g FW in mature plants [88]. In addition to its critical role in blood coagulation [87], phylloquinone also enhances bone quality by increasing strength and reducing turnover, ultimately lowering fracture rates [89].

Rapeseed (*B. napus*) oil is a key source of exogenous tocopherols, providing 37–51 mg/100 g of γ-tocopherol and 19–24 mg/100 g of α-tocopherol [90]. In *Brassica* vegetables, total tocopherol content ranges from 258.2 to 315.4 μg/g dry weight (DW) in broccoli and from 212.9 to 236.6 μg/g DW in cauliflower [91]. Tocopherols, derivatives of vitamin E, naturally occur in a wide variety of plants and vegetable oils. As potent plant-derived antioxidants, they not only promote skin health but also protect against harmful factors such as free radicals and ultraviolet radiation [92].

3)
**Carotenoids and Chlorophylls**

*Brassica* species are rich in carotenoids, compounds known for their health-promoting properties. Carotenoids, an important subgroup of isoprenoids, are widespread in nature and play crucial roles in human health, including antioxidant activity, immune system enhancement, and the well-known provitamin A activity [93]. Among the more than 700 carotenoids synthesized by plants, lycopene, β-carotene, β-cryptoxanthin, lutein, zeaxanthin, and α-carotene are the most significant and extensively studied dietary carotenoids. These compounds are present in various *Brassica* vegetables, though their content and composition vary [94] (Supplementary Table 1). Kale has been identified as having the highest total carotenoid content among vegetables at 13.3 mg per 100 g FW [72]. A detailed study on 33 kale cultivars revealed substantial intraspecific variation in carotenoid concentrations, ranging from 0.5 mg/g DW in wild types to 3.0 mg/g DW in the cultivated American “Champion” variety. Notably, zeaxanthin was the predominant carotenoid in 21 of these cultivars, with the highest concentrations found in the American varieties “Vates” and “Champion”, at 1.6 mg/g and 2.5 mg/g DW, respectively [95]. Crossbreeding appears to enhance carotenoid levels: in four out of five hybrid kale lines, zeaxanthin levels are significantly higher than in their parental lines, although β-carotene and lutein levels are lower, suggesting a strategic advantage of hybridization for boosting specific carotenoids [95]. The content of carotenoids also varies with the color of the vegetable. Guzman et al. [91] investigated various colored varieties of *B. oleracea*, including broccoli in green and purple, and cauliflower in white, purple, and orange. Notably, only white cauliflower lacked detectable levels of carotenoids. This is consistent with the color properties of carotenoids, underscoring the typical red, yellow, or orange hues associated with these compounds. By contrast, Chinese cabbage varieties with orange-colored inner leaves exhibited higher levels of lycopene-like compounds, such as prolycopene and its isomers, than those with yellow inner leaves ([96]; Zhang et al., 2015; [97]). Additionally, purple kohlrabi contains lower concentrations of carotenoids, such as β-carotene and lutein, compared to its pale green counterpart [68]. In headed cabbage, the levels of carotenoids (including neoxanthin, lutein, violaxanthin, and β-carotene) decrease from the outer to the inner layers [18]. Similar to that observed for vitamin C content, which ranges from 22.45–41.59 mg per 100 g FW, carotenoid content exhibits significant variations among different cultivars of leafy *B. rapa* subspecies, including pak choi, Chinese cabbage, mizuna, leafy turnip, tatsoi, ranging from 14.44 to 23.00 mg per 100 g FW (Artem’eva and Solov’eva, 2006). Interestingly, rectangular cultivars exhibit higher total carotenoid levels than their oval counterparts [69]. While zeaxanthin is the dominant carotenoid in kale [95], lutein is the most prevalent in Chinese cabbage, followed by β-carotene [69].

Lutein and zeaxanthin, components of the macular pigment, are crucial in preventing age-related macular degeneration, a neurodegenerative disease [98]. Contemporary research has revealed that various natural carotenoids, either alone or combined, play significant roles in cancer prevention. Multicarotenoid blends that include lycopene, α-carotene, β-carotene, and lutein demonstrate potent anti-carcinogenic properties [99]. Further studies reveal that when combined with α-tocopherol, these carotenoids notably reduce liver tumor growth in individuals with liver cirrhosis [99]. Consequently, the consumption of *Brassica* vegetables rich in carotenoids and tocopherols has reemerged as a biotherapeutic option, providing multiple health benefits by counteracting night blindness, slowing the progression of age-related macular degeneration, and reducing the incidence of lung and liver tumors.

Chlorophylls and their derivatives are pivotal photosynthetic pigments known for their broad spectrum of health-promoting effects, including antigenotoxic, antioxidant, anti-cancer, anti-obesogenic, and antimutagenic activities [100]. Chlorophyll levels vary significantly among different subspecies and cultivars of *Brassica* vegetables. Notably, four out of five hybrid kale lines exhibit lower levels of chlorophyll a and b compared to their parent lines [95]. In addition, all cauliflower types—whether white, purple, or orange—lack chlorophylls, while all three green broccoli types synthesize higher levels of chlorophyll than purple broccoli [91].

4)
**Phenolic compounds**

In addition to their rich vitamin and carotenoid contents, the potent antioxidant properties of *Brassica* vegetables are also attributed to their abundant phenolic composition. Polyphenol micronutrients have garnered increased interest for their protective properties against chronic diseases such as various cancers, cardiovascular disease, and type 2 diabetes [101–104]. Flavonoid, a predominant phenolic class in *Brassica* vegetables, exhibit considerable variation in content across types—from 15.3 mg per 100 g FW in white cabbage to 337.0 mg per 100 g FW in broccoli [105]—and subspecies—from 10.2 mg per 100 g FW in white cabbage to 94.4 mg per 100 g FW in Chinese cabbage [106]. Among 25 *B. oleracea* varieties, kale was noted to have the highest polyphenol concentration, at 27.0 mg per 100 g FW [72]. Red and green *B. oleracea* varieties are the richest in total phenolics and flavonoids, whereas white varieties have the lowest concentrations [72].

Anthocyanins, one of the most bioactive polyphenols, are notably abundant in red-pigmented *Brassica* varieties such as purple cauliflower (Li et al. 2012), red cabbage ([107]; Li et al. 2012), purple pak-choi [108], purple kohlrabi [68], and red curly kale [109]. By contrast, these compounds are present in very low concentrations in white and green *Brassica* varieties [72]. Specifically, anthocyanins are identified exclusively in the skin of purple kohlrabi, not flesh [68], and their levels decline from the outer to the inner layers in two green cabbage cultivars [18]. Dietary intake of anthocyanins is linked to reduced risks of cardiovascular and neurodegenerative diseases [101]. Additionally, phenolic fractions from *Brassica* vegetables have demonstrated antimicrobial effects against both gram-positive and gram-negative bacteria. Hydroalcoholic extracts of white cabbage and Brussels sprouts have been shown to inhibit the growth of *Listeria monocytogenes* [110], and ethanol extracts exhibit similar inhibitory activity against *Escherichia coli*, *B. subtillis*, and *S. epidermis* [111]. Furthermore, compared to pale green kohlrabi, purple kohlrabi exhibits higher levels of total phenylpropanoids [68].

5)
**Minerals**

*Brassica* vegetables are rich sources of essential minerals such as calcium, sodium, selenium, potassium, iron, and sulfur (Artem’eva and Solov’eva, 2006; [18]) (Supplementary Table 1). For instance, calcium levels in these crops range from 22 mg to 254 mg per 100 g FW, with kale exhibiting the highest levels (Supplementary Table 1). The bioavailability of calcium in these vegetables is particularly high due to their low levels of phytic acid and oxalate, compounds known to bind calcium into forms that are not absorbable by the body [112–114]. Additionally, *Brassica* vegetables are valued for their dietary potassium, which has been shown to help lower blood pressure, particularly in the context of a high-sodium diet [115]. Specifically, *B. rapa*, such as Chinese cabbage, is an excellent source of bioavailable calcium (averaging 1.40 mg/kg DW) and iron (averaging 558 mg/kg DW) [16]. In cabbage, concentrations of calcium, magnesium, sulfur, iron, and manganese decrease from the outer to inner leaves, whereas potassium and phosphorus are most concentrated in the 10th leaf layer [18]. Kale is distinguished among green leafy vegetables for its rich mineral content, which includes high levels of iron, calcium, potassium, phosphorus, and manganese, as well as notable quantities of zinc and selenium [116]. Despite these nutritional benefits, the consumption rate of kale in the US is relatively low compared to other *Brassica* vegetables, with per capita fresh intake of less than 0.33 kg per year, a figure that can be even lower in other countries due to varying dietary habits [117]. This limited intake restricts its impact on daily nutrition. Additionally, selenium (Se) has been recognized for its role in protecting against several common cancers, including lung, prostate, and colorectal cancers [118]. Se-containing amino acids might offer greater anticarcinogenic potential than standard sulfur-containing GSLs [119]. Although Se can be toxic to most plants [119], several *Brassica* vegetables, including canola (*B. napus*), Ethiopian mustard (*B. carinata*), broccoli (*B. oleracea*), Brussels sprouts (*B. oleracea*), and Indian mustard (*B. juncea*), are capable of accumulating unusually high levels of Se [70]. For example, broccoli or canola grown in Se-rich soils can accumulate up to 7 μg Se/g DW, significantly higher than the typical 0.1–0.3 μg Se/g DW [70, 120].

6)
**Edible *Brassica* vegetable parts and their nutritional value**

The nutritional value of plant-based foods is heavily influenced by the specific edible parts consumed and the methods of preparation. While most *Brassica* crops are eaten as fresh leafy vegetables, varieties like purple mustard (*B. juncea* (L.) Czern.), mizuna (*B. rapa* L. ssp. *nipposinica*), red cabbage (*B. oleracea* L. var. *capitata*), purple kohlrabi (*B. oleracea* L. var. *gongylodes*), and red mustard (*B. juncea* L. Czern.) are gaining popularity as microgreens. These seedlings are excellent dietary sources of antioxidants, including phylloquinone, ascorbic acid, tocopherols, and carotenoids [121]. For some *Brassica* crops, the seeds are used for vegetable oil extraction or condiment production. For example, the seeds of *B. carinata*, *B. juncea*, and *B. nigra* are used for mustard and mustard oil production [122]. Rapeseed oil prepared from *B. napus* is especially valued for its favorable nutritional profile, such as high levels of monounsaturated fatty acids, low levels of saturated fatty acids, and a favorable omega-3 fatty acid composition [123]. Its quality is further enhanced by the presence of phytosterols [123], carotenoids [124], erucic acid [125], choline, and tocopherols. The old cabbage variety *B. oleracea* L. var. *acephala* contains 94% unsaturated fatty acids but over 50% erucic acid in its seed oil [126]. Although this oil is rich in beneficial phytochemicals like γ-tocopherol, 11 polyphenol compounds, and 13 different carotenoids, the high erucic acid level raises significant health concerns [126].

### Impacts of processing and cooking methods on the nutritional composition of *brassica* vegetables

Processing methods such as boiling, steaming, stir-frying, and microwaving significantly affect the bioactive compounds (e.g. carotenoids, anthocyanins, and phenolic compounds) available for absorption by the human body [127]. In kale, all these processing methods reduce carotenoid contents but increase total phenolic levels, with steaming yielding the highest antioxidant activity and phenolic content [127]. However, in red cabbage, steaming leads to a 34.6% reduction in phenolic content [127]. A comprehensive study by Diamante et al. [128] investigated the effects of thermal processing—microwaving, boiling, and steaming—on the release of antioxidant compounds, carotenoids, and tocopherols in green, yellow, white, and purple cauliflowers, revealing that the impact of processing is largely independent of genotype. For example, boiling green cauliflower for 20 min resulted in the highest zeaxanthin (89.46 μg/100 g DW) and lutein (1563.71 μg/100 g DW) content [128]. The yellow variety showed the highest total carotenoid content in both raw (10.20 mg/100 g DW) and steamed (12.07 mg/100 g DW) forms, with the latter also exhibiting the highest total carotene levels (11.26 mg/100 g DW). Furthermore, microwaving and boiling purple cauliflower for extended periods retained the highest levels of tocopherols [128].

Phan et al. [129] reported enhanced anti-inflammatory and antioxidant effects at the cellular level in red cabbage co-digested with vegetables like baby spinach, carrots, and cherry tomatoes, compared to the digestion of these vegetables individually. Notably, co-digestion of red cabbage with baby spinach was especially beneficial, resulting in high bioaccessible carotenoid levels and demonstrating synergistic effects across all tested bioactivities. These included reduced secretion of cellular interleukin-8 and nitric oxide, as well as enhanced antioxidation [129]. Phylloquinone, a lipophilic compound, is co-extracted with vegetable oils, making them an important dietary source of vitamin K [87]. Interestingly, no significant difference was observed in phylloquinone absorption between consuming fresh or cooked broccoli in meals containing 30% or 45% energy from fat [130]. The antioxidant capacity of *Brassica* vegetables is affected by processing, with qualitative changes, thermal breakdown, and the leaching of compounds into water during cooking [105]. Therefore, consuming raw *Brassica* vegetables with a small amount of vegetable oil or fat is the optimal approach to maximize antioxidant nutrient intake.

### Biofortification and nutritional enhancement of *brassica* vegetables

**Figure 3:**
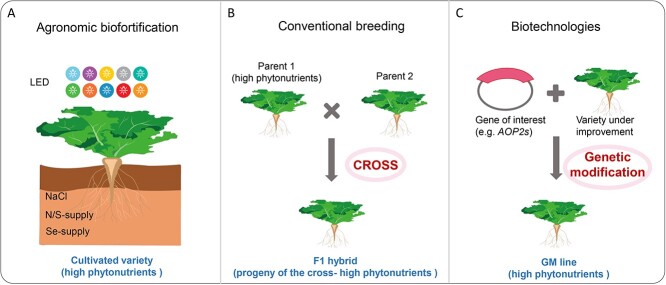
Biofortification and nutritional enhancement of *Brassica* vegetables achieved through (A) agronomic practices, (B) conventional breeding, and (C) plant biotechnologies. N, nitrogen; S, sulfur; Se, selenium; GM, genetic modification.

As a group of widely consumed vegetables with increasing popularity, *Brassica* vegetables have demonstrated their capability and significant potential to contribute to dietary nutrition. Unlike staple crops, these vegetables offer distinct health advantages due to their inherent nutritional benefits. Two emblematic Brassica vegetables kale and broccoli are widely recognized as “superfoods” or “functional foods” due to their exceptional nutritional values [131, 132]. The accumulation of bioactive compounds in these vegetables is influenced by both external factors such as cultivation conditions and internal factors such as genetic variations (Fig. 3). Biofortification—the process of enhancing the mineral and vitamin content in crops—can be effectively pursued through agronomic practices, biotechnological approaches, and plant breeding.

1)
**Agronomic biofortification**

Agronomic biofortification has been explored extensively in *Brassica* crops, achieving notable success (Fig. 3A). The density and composition of light profoundly affect plant development, growth, and metabolic profiles, thereby affecting nutritional values. For example, a higher percentage of blue light has been shown to promote the growth of carotenoid-rich sprouts in pak choi (*B. rapa* ssp. *chinensis* “Black Behi”) [133]. Specifically, using a combination of blue and white LEDs increases the carotenoid content by ~15%, whereas a combination of red and white LEDs reduces it [133]. *Brassica* sprouts are becoming increasingly popular as a fresh and nutritious food choice due to their low levels of antinutritional components and high content of dietary fiber and micronutrients [134]. Since *Brassica* sprouts are commonly cultivated in controlled environments, the growing conditions are crucial in determining their nutritional value. Vale et al. [135] demonstrated that dark conditions enhance the nutritional quality of *B. oleracea* sprouts, increasing dietary fiber, minerals, and fatty acid contents. Conversely, exposure to light increases the content of selenium, a mineral known for its health benefits. Exposure to enhanced light intensity across various wavelengths (white, red, far-red, and blue) leads to increased pigment accumulation in red Russian kale (*B. napus*) seedlings, including chlorophyll and anthocyanins. However, these seedlings are particularly sensitive to far-red light, resulting in elevated levels of chlorophyll and anthocyanins even under very low light fluence rates of less than 1 μmol m^−2^ s^−1^ [136].

Light conditions also influence glucosinolate content in *Brassica* vegetables. For instance, far-red light has been found to increase aliphatic GSLs in red Russian kale sprouts, whereas sequential exposures to far-red, red, and blue light, as well as periods of darkness, can alter their metabolic and nutrient profiles [136]. In summary, LEDs offer an effective means to regulate phytonutrient contents, including carotenoids, selenium, chlorophyll, and anthocyanins, in *Brassica* sprouts. In contrast, a 2-year study comparing conventional and organic cultivation systems found that organically grown fresh cabbage contained significantly lower levels of total flavonoids, total chlorophylls, nitrites, and nitrates. However, organic sauerkraut juice has notably higher levels of total sugars and polyphenols [137]. Interestingly, the total polyphenols in red cabbage, white cabbage, and Brussels sprouts showed no significant differences across three diverse environments: near a steelworks, on an organic farm, and at a market [138]. Interactive effects analysis of N/S-supply on GSLs in two Chinese cabbage cultivars revealed that GSL profiles are primarily influenced by the genetic background of the cultivar, followed by fertilizers [139]. The concentration of sodium chloride (NaCl) also impacts glucosinolate content and composition; 50 mM NaCl, for instance, significantly increases total GSLs, two aliphatic GSLs (GAL and gluconapin, GNP) and two indole GSLs (GBS and NGBS) in pak choi [140]. Pre- and post-harvest practices can further optimize GSL content [141]. When grown in selenium-enriched soils, *Brassica* species such as Indian mustard (*B. juncea*), Ethiopian mustard (*B. carinata*), canola (*B. napus*), and various *B. oleracea* vegetables can accumulate selenium at levels significantly above normal, which facilitates the synthesis of active organic selenium compounds, such as seleno-amino acids, from inorganic sources [70, 119, 120]. Enriching the soil with selenium where *Brassica* vegetables are cultivated can significantly enhance the dietary intake of this crucial mineral. Selenium is absorbed by plants through the sulfur (S) pathway, and studies have shown that a combination of 4 mmol/L sulfur and 100 μmol/L selenium substantially increases the yield of SFN, enhances myrosinase activity, and raises Se-methylselenocysteine levels in broccoli [119, 142, 143].

2)
**Conventional breeding**

Biofortification through conventional breeding activities has proven effective, particularly with staple crops [144]. This classical genetic approach can also create new vegetable food sources enriched with high concentrations of natural phytonutrients, achieved through the crossing of cultivars or plants already abundant in these metabolites (Fig. 3B). The vast genetic diversity within the germplasm, natural variations, and breeding populations of *Brassica* crops distributed globally provide invaluable resources with considerable potential for developing varieties with enhanced nutritional value [145]. An example of such success is the creation of the high-GRA “super broccoli”, which was developed through traditional breeding methods [146]. Additionally, Mageney et al. [95] reported that four out of five hybrid kale lines (*B. oleracea* var. *sabellica*) had significantly higher zeaxanthin levels compared to their parent lines, underscoring the potential of crossbreeding to boost carotenoid levels in kale. Furthermore, substantial natural genetic variation and heritability in calcium Ca^2+^ and Mg^2+^ levels have been observed in *B. rapa* and *B. oleracea*, suggesting that it is feasible to enhance these essential minerals in *Brassica* vegetables [147].

3)
**Biotechnologies and metabolic engineering**

Compared to the prolonged timelines associated with traditional breeding, biofortification through biotechnologies and metabolic engineering offers significant advantages for rapidly enhancing the nutritional value of a wide range of crops [148]. Advances in understanding the mechanistic underpinnings of the biosynthesis, regulation, transport, storage, and degradation of secondary metabolites that confer health benefits have greatly facilitated the development of nutrient-enriched *Brassica* crops. This process can be expedited using cutting-edge biotechnologies such as CRISPR/Cas9-mediated genome editing [149] (Fig. 3C). Given that the genomes of Brassica species have undergone whole genome triplication, leading to the expansion of gene families and the creation of multiple gene copies, exploring trait diversity in these crops presents more complexity than in simpler models like *Arabidopsis* [150].

**i Glucosinolates**

GSLs, key secondary metabolites in *Brassica* crops, contribute significantly to their nutritional value. The biosynthetic and regulatory pathways of GSLs, as well as related biofortification strategies, have been extensively studied. Miao et al. [151] recently reviewed methods for enhancing GSLs in *Brassica* crops through metabolic engineering. These methods include the ablation of hydrolysis, modulation of biosynthesis, redirection of metabolic flux, inhibition of transport, and manipulation of regulatory proteins. For example, the overexpression of *flavin-containing monooxygenase* (*FMO_GS-OX_*) genes in turnip notably increases aliphatic glucosinolate levels in transgenic turnip hairy roots [152]. Additionally, BoRHON1, a newly identified RNA-binding protein, acts as an upstream regulator of *BoMYB28–3*, the principal transcriptional regulator of aliphatic glucosinolate biosynthesis in cabbage [153]. Overexpression of BoRHON1 in *Arabidopsis* induces the accumulation of both aliphatic and indolic GSLs [153]. Additionally, omics data analysis revealed that turnip possesses the most functional *methylthioalkylmalate synthase* (*MAM*) genes (at least five *MAM*s), whereas the other *Brassica* species/subspecies, such as Chinese cabbage, pakchoi, cabbage, and *B. nigra*, typically contain only 1–3 *MAM* pseudogenes. This leads to distinct aliphatic GSL profiles in Chinese cabbage and turnip [14]. In broccoli, the role of MAM1 protein in diversifying GSL biosynthesis has been documented [154]. Four BoMAM proteins (BolI0108790, BolI0090270, BolI0088930 and BolI0108770) have been identified as variants of BoMAM1. Notably, overexpression of *BoMAM1* (BolI0108790) in broccoli leads to a significant increase in the accumulation of C4-GSLs, including GER, GRA, GNP, and PRO [154]. Additionally, transposable element insertions in one MAM3 gene copy impact long chain aliphatic GSL accumulation in broccoli, cauliflower, and kohlrabi [155]. The silencing of *2-oxoglutarate-dependent dioxygenase* (*AOP2*) genes through RNA interference decreases levels of the detrimental PRO compounds but notably increases contents of the desirable GRA in *B. juncea* and *B. napus* seeds [156, 157]. Further, the introgression of non-functional *braop2.2* and *braop2.3* alleles result in increased levels of beneficial GRAs in *B. rapa* [158]. Typically, three active *AOP2* genes are present in most *Brassica* species/subspecies, but in *B. oleracea*, only one of these genes is functional, which may explain the elevated GRA levels observed in this species [14]. Additionally, the non-functionality of *BoAOP2* in broccoli accounts for the unique accumulation of two health-promoting GSLs, GRA and glucoalyssin [155]. These findings suggest that gene-editing technologies such as CRISPR-Cas9 could be strategically employed to manipulate *AOP2* genes, thus potentially increasing beneficial GRA while reducing harmful PRO compounds in *Brassica* vegetables [67]. By increasing *MAM* expression and silencing *AOP2*, it is possible to enhance the accumulation of anticancer GSLs, including GRA.

**ii Carotenoids.**

Metabolic engineering offers two primary strategies for enhancing carotenoids in crops: increasing synthesis and storage (“pull”) and reducing degradation (“protect”). A thorough understanding of the molecular mechanisms involved is crucial for devising effective solutions. For instance, higher transcript levels of carotenoid biosynthesis genes such as *BrPSY*, *BrZEP*, *BrPDS*, *BrLCYE*, *BrZDS*, *BrCHXB*, and *BrLCYB* are correlated with increased carotenoid content in Chinese cabbage [159]. The *Or* gene enhances the accumulation of carotenoids, particularly β-carotene, by inducing chromoplast formation rather than directly regulating their biosynthesis. This gene causes unpigmented tissues, such as the curd, shoot meristems, pith, and leaf bases, in cauliflower to turn orange, a phenotype also observed in orange *Or* transgenic potato tubers [160, 161]. Conversely, a recessive mutation in the *Br-or* gene, a carotenoid isomerase known as *BrCRTISO*, leads to the development of orange leafy heads, flowers, and cotyledons in Chinese cabbage rich in lycopene-like compounds, specifically prolycopene and its isomers ([96, 162, 163]; Zhang et al., 2015). Natural variations in the *BrHISN2* promoter lead to decreased chlorophyll content and a distinct flavor profile in Chinese cabbage with yellow leafy heads, which is preferred by consumers. This phenotype is characterized by lower levels of cellulose, sugar, and soluble protein [164]. In flowering Chinese cabbage (Caixin, *B. rapa* L. ssp. *chinensis var. parachinensis*), a 1148 bp deletion in the promoter of the *PALE YELLOW PETAL* (*BrPYP*) gene, which encodes a phytyl ester synthase 2 protein, results in pale-yellow petal colors due to reduced levels of esterified carotenoids [165]. Additionally, increased expression of the *PSY* gene is associated with enhanced carotenoid formation in yellow turnip [166]. These genetic loci present valuable targets for metabolic engineering aimed at boosting carotenoid levels. For example, in *B. napus* seeds, reducing the expression of *lycopene ε-cyclase* (*ε-CYC*) leads to increased levels of total carotenoids, including violaxanthin, β-carotene, lutein, and zeaxanthin, demonstrating the potential for genetic interventions to improve nutritional content [124].

**iii Flavonoids.**

In kohlrabi, substantial increases in the expression levels of the anthocyanin biosynthetic gene *BoF3H* and two regulatory genes, *BoTT8* and *BoPAP2*, are observed in the purple skin, leaves, and swollen stems (Zhang et al., 2015). A similar pattern is observed in Chinese cabbage, where the integration of anthocyanin content and gene expression analyses suggests that *BrMYB2* and *BrTT8* potentially activate anthocyanin biosynthesis in the purple leaves of the heading [167]. In pak choi, the dominant gene *BrPur* plays a critical role in regulating the accumulation of anthocyanins in the epidermis and adjacent mesophyll cells [108]. Similarly, in purple-leaf mustard (*B. juncea*), increased transcription of *BjPur*, which encodes an R2R3-MYB transcription factor, is associated with increased anthocyanin accumulation [168]. Additionally, overexpression of the *Arabidopsis Production of Anthocyanin Pigment 1* (*AtPAP1*) gene in canola results in increased levels of anthocyanins, flavonoids, and phenolics, leading to purple-green leaves and stems [169]. Moreover, in *B. napus*, the adenosine 5′-phosphosulfate reductase gene is negatively associated with anthocyanin accumulation in leaves, indicating a negative regulatory role [170]. The transporter gene *BrTT19*, as well as two regulatory genes *BolTT8* and *BrMYB111*, are crucial for anthocyanin production and accumulation in resynthesized *B. napus* derived from its two diploid parents *B. oleracea* and *B. rapa* [171].

Furthermore, *BnaPAP2.A7*, an ortholog of the *B. oleracea* anthocyanin activator *BoMYB2*, imparts purple traits to cauliflower, and its high expression level is crucial for driving anthocyanin biosynthesis in *B. napus* leaves [172, 173]. Other *R2R3-MYB* genes, such as *BnaC06.PAP2* and *BnaA07.PAP2*, also contribute to anthocyanin biosynthesis [174]. Finally, the flavonol synthases (FLSs) BnaFLS1–2 and BnaFLS1–1 in *B. napus* possess activities of the FLS and flavanone 3-hydroxylase (F3H) enzymes, whereas BnaFLS3–4 and BnaFLS3–3 only exhibit F3H activity, which is essential for flavonol formation [175].

**iv Others**

Variations in the *fatty acid elongase1* (*FAE1*) gene are closely linked to erucic acid synthesis in *Brassica* seeds. Specific SNPs/indels (insertions/deletions) in *FAE1-A8* and *FAE1-C3* are associated with reduced erucic acid content in the seeds of *B. napus* and *B. rapa*, respectively [176]. Additionally, overexpression of the orphan gene *BrOG1* in *Brassica* significantly increases the contents of glucose, fructose, and total sugars while reducing sucrose levels [177].

Knowledge gained from studying the biosynthetic pathways of key micronutrients will further facilitate the breeding of nutrient-enriched *Brassica* crops (Table 3), such as utilizing SNP markers of FAE1 to select for reduced erucic acid content in seeds [176]. Advanced genetic technologies, such as genome editing, offer promising strategies to boost the nutritional value of *Brassica* crops, thereby supporting the development of healthier food options for a growing global population [180]. For example, enhancing *MAM* expression while reducing *AOP2* expression not only promotes the accumulation of the anticancer GRA but also inhibits the synthesis of harmful PRO. Additionally, leveraging the dominant *Or* gene in cauliflower and the recessive mutation of *Br*-*or* in Chinese cabbage enhances the accumulation of carotenoids in the tissues (e.g. the curd and pith) and orange leafy heads, respectively [96, 160–163, 179]. Furthermore, the upregulation of R2R3-MYB transcription factors has proven to be an effective method for improving anthocyanin synthesis in *Brassicas* [167, 168, 171–174].

**Table 3:** Genes responsible for phytonutrients synthesis or accumulation in *Brassica* vegetables

Phytonutrients	Genes	References
Glucosinolates	*MAMs*	[14]; [154]; [155]
	*AOP2s*	[157]; [156]; [158]; [67]; [14]; [155]
	*FMO_GS-OX_*	[152]
	BoRHON1*-MYB28*	[153]
Carotenoids	*Or*	[160]; [161]
	*BrCRTISO*	[162]; [163]; [96]; [178]
	*ε-CYC*	[124]
	*BrPYP*	[165]
	*PSY*	[166]
Chlorophylls	*BrHISN2*	[164]
Flavonoids	*TT8*	[179]; [167]; [171]
	*F3H*	[179]
	*PAP2*	[178]; [172]; [174]
	*MYB2*	[173]; [167]
	*Pur*	[108]; [168]
	*APR2*	[170]
	*MYB111*	[171]
	*TT19*	[171]
	FLS1s/3 s	[175]
	*AtPAP1*	[169]
Erucic acid	*FAE1*	[176]

## Conclusion and perspectives

Including *Brassica* vegetables in the diet provides a broader and more varied array of phytonutrients compared to pharmaceuticals, including glucosinolates, vitamins (C, tocopherol, folate, and phylloquinone), carotenoids, phenolics, and minerals. It is often overlooked that many medications originate from natural compounds initially found in *Brassica* foods [80]. *Brassica* vegetables are considered “functional foods” due to their potential health benefits in preventing chronic diseases such as cardiovascular disease, type 2 diabetes, and cancer [105, 181]. Consequently, the development of fruits and vegetables with enhanced medicinal or nutritional properties is increasingly becoming a major focus of breeding programs [182]. The carotenoid and anthocyanin enhancement strategies in commercial tomato lines provide useful insights into nutritional fortification in vegetables [183, 184]. Orange and purple tomatoes rich in carotenoids and anthocyanins have been successfully developed using both transgenic and conventional breeding techniques [183, 184]. In *Brassica*s, the genes associated with the accumulation of health-promoting compounds offer opportunities to enhance bioactive nutrients through a combination of conventional breeding and metabolic engineering. Additionally, the use of LEDs (white, blue, far-red) and selenium-enriched cultures provides effective agronomic methods to increase the concentration of phytonutrients in *Brassica* sprouts. Nevertheless, *Brassica* vegetables remain a largely untapped reservoir of healthy nutrients that merits further exploration. Ongoing research is essential to unravel the genetic mechanisms behind phytonutrient synthesis and accumulation, which will guide the biofortification and metabolic engineering efforts aimed at boosting the nutritional profile of *Brassica* crops. Notably, recent studies have discovered novel functional nutrients, such as γ-aminobutyric acid, in *Brassica* vegetables like Chinese cabbage and *B. napus* seedlings, which are important for brain plasticity and are linked to neurological and psychiatric disorders [185, 186]. In summary, this review underscores the rich phytochemicals and health benefits of *Brassica* crops and outlines strategies aimed at enhancing their nutritional value, suggesting a promising direction for future agricultural innovation and dietary health improvements.

## Supplementary Material

Web_Material_uhae302
