# Supplementary material for: Brassica vegetables—an undervalued nutritional goldmine
Source: Hortic Res. 2024 Oct 30;12(2):uhae302. doi: 10.1093/hr/uhae302 (PMC11822409; doi:10.1093/hr/uhae302)
Supplement: Web_Material_uhae302 [file web_material_uhae302.zip › Supplementary Figure 1 0522.docx]

***Brassica* vegetables - An undervalued nutritional goldmine**

Xiaomeng Zhang^1, +^, Qiong Jia^1, +^, Xin Jia^1, +^, Jie Li^2^, Xiaoxue Sun^1^, Leiguo Min^1^, Zhaokun Liu^3^, Wei Ma^1, *^, Jianjun Zhao^1, *^

^1^State Key Laboratory of North China Crop Improvement and Regulation, Key Laboratory of Vegetable Germplasm Innovation and Utilization of Hebei, Collaborative Innovation Center of Vegetable Industry in Hebei, College of Horticulture, Hebei Agricultural University, 071000 Baoding, China.

^2^John Innes Centre, Norwich Research Park, Norwich, UK.

^3^Suzhou Academy of Agricultural Sciences, Suzhou, Jiangsu 215155, China.

^+^These authors contributed equally to this work.

^*^Correspondence should be addressed to Jianjun Zhao (yyzjj@hebau.edu.cn) and Wei Ma (yymw@hebau.edu.cn), Tel.: +86 (0312)7528323, Fax: 03127521287.

The email addresses of other authors:

Xiaomeng Zhang, 13716247546@163.com

Qiong Jia, 17731245331@163.com

Xin Jia, 15831273770@163.com

Jie Li, jie.li@jic.ac.uk

Leiguo Min, m17609360250@icloud.com

Zhaokun Liu, saaslzk@qq.com


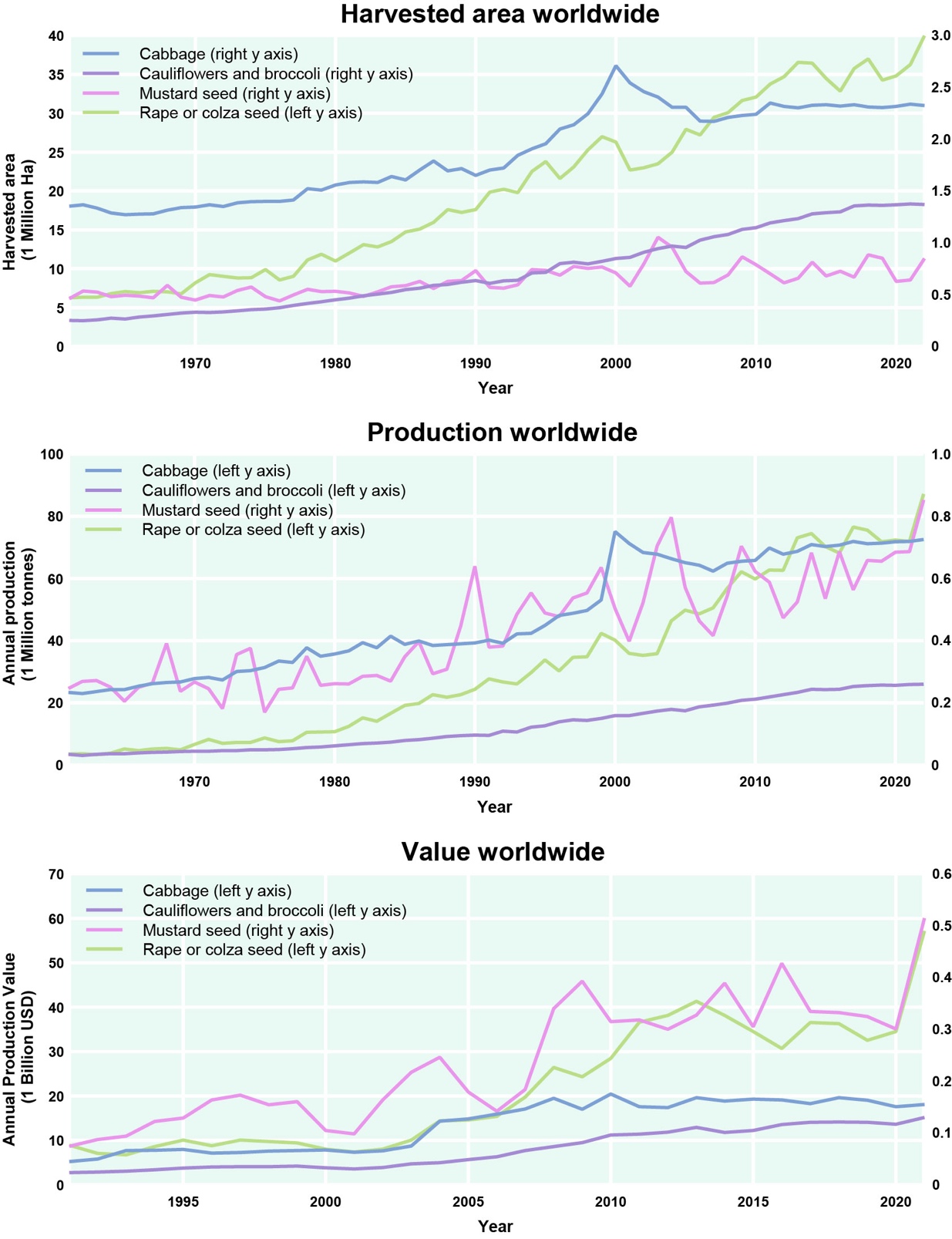


Supplementary Figure 1. Harvested area, production and value (from top to bottom) of major *Brassica* crops increased from 1961 to 2022. Data source: Food and Agriculture Organization (FAO).
