# Supplementary material for: Brassica vegetables—an undervalued nutritional goldmine
Source: Hortic Res. 2024 Oct 30;12(2):uhae302. doi: 10.1093/hr/uhae302 (PMC11822409; doi:10.1093/hr/uhae302)
Supplement: Web_Material_uhae302 [file web_material_uhae302.zip › Supplementary_table_1.doc]

| **Supplementary Table 1. Nutritional value of *Brassica* vegetabels (100 g fresh weight)** | | | | | | | | | | | | | | | |
| --- | --- | --- | --- | --- | --- | --- | --- | --- | --- | --- | --- | --- | --- | --- | --- |
|  | **RDA** | | ***Brassica rapa*** | | | ***Brassica oleracea*** | | | | | | | ***Brassica napus*** | ***Brassica juncea*** | **Data Source** |
|  | **Male** | **Female** | **Chinese cabbage** | **Pak choi** | **Turnip** | **Broccoli** | **Cauliflower** | **Kohlrabi** | **Kale** | **Cabbage** | **Red cabbage** | **Brussels sprouts** | **Rutabagas** | **Mustard greens** |  |
| Water (g) |  |  | 94.4 | 95.3 | 91.9 | 89.3 | 92.1 | 91 | 89.6 | 92.2 | 90.4 | 86 | 89.4 | 90.7 | USDA FoodData Central |
| Energy (kal) | 2500 | 2000 | 16 | 13 | 28 | 34 | 25 | 27 | 35 | 25 | 31 | 43 | 37 | 27 | USDA FoodData Central |
| Protein (g) | 55.5 | 45 | 1.2 | 1.5 | 0.9 | 2.82 | 1.92 | 1.7 | 2.92 | 1.28 | 1.43 | 3.38 | 1.08 | 2.86 | USDA FoodData Central |
| Total fat (g) | 97 | 78 | 0.2 | 0.2 | 0.1 | 0.37 | 0.28 | 0.1 | 1.49 | 0.1 | 0.16 | 0.3 | 0.16 | 0.42 | USDA FoodData Central |
| Carbohydrate (g) | 333 | 267 | 3.23 | 2.18 | 6.43 | 6.64 | 4.97 | 6.2 | 4.42 | 5.8 | 7.37 | 8.95 | 8.62 | 4.67 | USDA FoodData Central |
| Dietary fiber (g) | 30 | 30 | 1.2 | 1 | 1.8 | 2.6 | 2 | 3.6 | 4.1 | 2.5 | 2.1 | 3.8 | 2.3 | 3.2 | USDA FoodData Central |
| Total Sugars (g) | 33 | 27 | 1.41 | 1.18 | 3.8 | 1.7 | 1.91 | 2.6 | 0.99 | 3.2 | 3.83 | 2.2 | 4.46 | 1.32 | USDA FoodData Central |
| ***Minerals*** |  |  |  |  |  |  |  |  |  |  |  |  |  |  |  |
| Calcium (mg) | 700 | 700 | 77 | 105 | 30 | 47 | 22 | 24 | 254 | 40 | 45 | 42 | 43 | 115 | USDA FoodData Central |
| Iron (mg) | 8.7 | 14.8(19-50y)  8.7 (50-64y) | 0.31 | 0.8 | 03 | 0.73 | 0.42 | 0.4 | 1.6 | 0.47 | 0.8 | 1.4 | 0.44 | 1.64 | USDA FoodData Central |
| Magnesium (mg) | 300 | 270 | 13 | 19 | 11 | 21 | 15 | 19 | 33 | 12 | 16 | 23 | 20 | 32 | USDA FoodData Central |
| Phosphorus (mg) | 550 | 550 | 29 | 37 | 27 | 66 | 44 | 46 | 55 | 26 | 30 | 69 | 53 | 58 | USDA FoodData Central |
| Potassium (mg) | 3500 | 3500 | 238 | 252 | 191 | 316 | 299 | 350 | 348 | 170 | 243 | 389 | 305 | 384 | USDA FoodData Central |
| Sodium (mg) | 2400 | 2400 | 9 | 65 | 67 | 33 | 30 | 20 | 53 | 18 | 27 | 25 | 12 | 20 | USDA FoodData Central |
| Zinc (mg) | 9.5 | 7 | 0.23 | 0.19 | 0.27 | 0.41 | 0.27 | 0.03 | 0.39 | 0.18 | 0.22 | 0.42 | 0.24 | 0.25 | USDA FoodData Central |
| Copper (mg) | 1.2 | 1.2 | 0.036 | 0.021 | 0.085 | 0.049 | 0.039 | 0.129 | 0.053 | 0.019 | 0.017 | 0.07 | 0.032 | 0.165 | USDA FoodData Central |
| Manganese (mg) | 2.3 | 1.8 | 0.19 | 0.159 | 0.134 | 0.21 | 0.155 | 0.139 | 0.92 | 0.16 | 0.243 | 0.337 | 0.131 | na | USDA FoodData Central |
| Selenium (µg) | 75 | 60 | 0.6 | 0.5 | 0.7 | 2.5 | 0.6 | 0.7 | 0.9 | 0.3 | 0.6 | 1.6 | 0.7 | 0.9 | USDA FoodData Central |
| ***Vitamins*** |  |  |  |  |  |  |  |  |  |  |  |  |  |  |  |
| Vitamin A (µg) | 700 | 600 | 16 | 223 | 0 | 31 | 0 | 2 | 241 | 5 | 56 | 38 | 0 | 151 | USDA FoodData Central |
| Thiamin (mg) | 1 | 0.8 | 0.04 | 0.04 | 0.04 | 0.071 | 0.05 | 0.05 | 0.113 | 0.061 | 0.064 | 0.139 | 0.09 | 0.08 | USDA FoodData Central |
| Riboflavin (mg) | 1.3 | 1.1 | 0.05 | 0.07 | 0.03 | 0.117 | 0.06 | 0.02 | 0.347 | 0.04 | 0.069 | 0.09 | 0.04 | 0.11 | USDA FoodData Central |
| Niacin (mg) | 16.5 | 13.2 | 0.4 | 0.5 | 0.4 | 0.639 | 0.507 | 0.4 | 1.18 | 0.234 | 0.418 | 0.745 | 0.7 | 0.8 | USDA FoodData Central |
| Pantothenic acid (mg) | 5 | 5 | 0.105 | 0.088 | 0.2 | 0.573 | 0.667 | 0.165 | 0.37 | 0.212 | 0.147 | 0.309 | 0.16 | 0.21 | USDA FoodData Central |
| Vitamin B6 (mg) | 1.4 | 1.2 | 0.232 | 0.194 | 0.09 | 0.175 | 0.184 | 0.15 | 0.147 | 0.124 | 0.209 | 0.219 | 0.1 | 0.18 | USDA FoodData Central |
| Folate (µg) | 200 | 200 | 79 | 66 | 15 | 63 | 57 | 16 | 62 | 43 | 18 | 61 | 21 | 12 | USDA FoodData Central |
| Vitamin B12 (µg) | 1.5 | 1.5 | 0 | 0 | 0 | 0 | 0 | 0 | 0 | 0 | 0 | 0 | 0 | 0 | USDA FoodData Central |
| Vitamin C (mg) | 40 | 40 | 27 | 45 | 21 | 89.2 | 48.2 | 62 | 93.4 | 36.6 | 57 | 85 | 25 | 70 | USDA FoodData Central |
| Vitamin E (mg) | 4 | 3 | 0.12 | 0.09 | 0.03 | 0.78 | 0.08 | 0.48 | 0.66 | 0.15 | 0.11 | 0.88 | 0.3 | 2.01 | USDA FoodData Central |
| Vitamin D (µg) | 10 | 10 | 0 | 0 | 0 | 0 | 0 | 0 | 0 | 0 | 0 | 0 | 0 | 0 | USDA FoodData Central |
| Vitamin K (phylloquinone) (µg) | 120 | 90 | 42.9 | 45.5 | 0.1 | 102 | 15.5 | 0.1 | 390 | 76 | 38.2 | 177 | 0.3 | 258 | USDA FoodData Central |
| ***Other phytonutrients*** |  |  |  |  |  |  |  |  |  |  |  |  |  |  |  |
| ***Carotenoids*** |  |  |  |  |  |  |  |  |  |  |  |  |  |  |  |
| β-Carotene (µg) |  |  | 190 | 2680 | 0 | 361 | 0 | 22 | 2870 | 42 | 670 | 450 | 1 | 1790 | USDA FoodData Central |
| α-Carotene (µg) |  |  | 1 | 1 | 0 | 25 | 0 | 0 | 0 | 33 | 0 | 6 | 0 | 10 | USDA FoodData Central |
| β-Cryptoxanthin (µg) |  |  | 0 | 0 | 0 | 1 | 0 | 0 | 27 | 0 | 0 | 0 | 1 | 40 | USDA FoodData Central |
| Lycopene (µg) |  |  | 0 | 0 | 0 | 0 | 0 | 0 | 0 | 0 | 20 | 0 | 14 | 0 | USDA FoodData Central |
| Lutein + zeaxanthin (µg) |  |  | 48 | 40 | 0 | 1400 | 1 | 0 | 6260 | 30 | 329 | 1590 | 19 | 3730 | USDA FoodData Central |
| Choline (mg) | 550 | 425 | 7.6 | 6.4 | 11.1 | 18.7 | 44.3 | 12.3 | 0.5 | 10.7 | 17.1 | 19.1 | 14.1 | 0.5 | USDA FoodData Central |
| ***Flavonoids*** |  |  |  |  |  |  |  |  |  |  |  |  |  |  |  |
| Quercetin (mg) |  |  | 39^a^ | 2.06^b^ | 0.73^b^ | 13.7^a^ | 3.9^a^ | 0.4^b^ | 22.58^b^ | 5.1^a^ | 0.36^b^ | 1.92^b^ | 0.05^b^ | 8.8^b^ | a: Bahorun et al 2004; b: Bhagwat et al 2014  c: Chen et al 2008; d: Linus Pauling Institute Micronutrient Center |
| Kaempferol (mg) |  |  | 9.6^a^ | 4.33^b^ | 11.87^b^ | 4.6^a^ | 1.2^a^ | 2.43^b^ | 46.8^b^ | 0^a^ | 0^b^ | 0.86^b^ | 0.32^b^ | 38.3^b^ |  |
| Apigenin (mg) |  |  | 4.5^a^ | 0.24^b^ | 0^b^ | 0^a^ | 0.2^a^ | 0^b^ | 0^b^ | 0.8^a^ | 0.06^b^ | 0^b^ | 3.85^b^ | N.D |  |
| Luteolin (mg) |  |  | 1.2^a^ | 0.09^b^ | 0^b^ | 0^a^ | 0^a^ | 1.3^b^ | 0^b^ | 0^a^ | 0.1^b^ | 0.33^b^ | 0^b^ | N.D |  |
| Cyanidin (mg) |  |  | N.D | N.D | N.D | 0^b^ | N.D | N.D | N.D | N.D | 209.83^b^ | N.D | N.D | N.D |  |
| Total Glucosinolates (mg) |  |  | 10-35^c^ | 54.28^d^ | 92.31^d^ | 61.36^d^ | 44^d^ | 42.27^d^ | 100^d^ | 77.78^d^ | 64.44^d^ | 236.36^d^ | N.D | 282.14^d^ |  |
| RDA, recommended daily intake for adults aged 19 to 64; N.D, no data. | | | | | | | | | | | | | |  |  |
|  | | | | | | | | | | | | | |  |  |
| a | Bahorun T, Luximon‐Ramma A, Crozier A, Aruoma OI. Total phenol, flavonoid, proanthocyanidin and vitamin C levels and antioxidant activities of Mauritian vegetables. Journal of the Science of Food and Agriculture. 2004, 84(12): 1553-1561. | | | | | | | | | | | | | | |
| b | Bhagwat, S., Haytowitz, D.B. Holden, J.M. (Ret.). 2014. USDA Database for the Flavonoid Content of Selected Foods, Release 3.1. U.S. Department of Agriculture, Agricultural Research Service. Nutrient Data Laboratory Home Page: http://www.ars.usda.gov/nutrientdata/flav. | | | | | | | | | | | | | | |
| c | Chen X, Zhu Z, Gerendás J, Zimmermann N. Glucosinolates in Chinese Brassica campestris Vegetables: Chinese Cabbage, Purple Cai-tai, Choysum, Pakchoi, and Turnip. HortScience horts. 2008, 43(2): 571-574. | | | | | | | | | | | | | | |
| d | Higdon J 2005. Linus Pauling Institute Micronutrient Center 2006, updated 2005: https://lpi.oregonstate.edu/mic/dietary-factors/phytochemicals/isothiocyanates. | | | | | | | | | | | | | | |
|  |  | | | | | | | | | | | | | | |
